# Climate change distress and impairment in Germany

**DOI:** 10.3389/fpubh.2024.1432881

**Published:** 2024-09-24

**Authors:** Lars König, Priska Breves, Gesa Alena Linnemann, Tim Hamer, Ralf Suhr

**Affiliations:** ^1^Stiftung Gesundheitswissen, Berlin, Germany; ^2^Institut für Medizinische Soziologie und Rehabilitationswissenschaft, Charité – Universitätsmedizin Berlin, Berlin, Germany; ^3^Amsterdam School of Communication Research, University of Amsterdam, Amsterdam, Netherlands; ^4^Fachbereich Gesundheit, Katholische Hochschule Nordrhein-Westfalen, Cologne, Germany

**Keywords:** climate change, distress, Germany, health, impairment, mental health, representative, survey

## Abstract

**Introduction:**

Climate change has been widely recognized as one of the most challenging problems facing humanity and it imposes serious mental health threats. It is important, however, to differentiate between the affective experience of distress over climate change and the functional impairments associated with climate change. Such a distinction is crucial because not all negative affective states are pathological, and they might even motivate pro-environmental behavior. Functional impairments, like not being able to work or maintaining social relationships, however, might require immediate treatment. This study assesses climate change distress and climate change impairment within the population of Germany using a population-representative sample. The results identify vulnerable subgroups and thereby can help to facilitate the development of target group specific intervention programs. Furthermore, this study explores whether climate change distress and climate change impairment are associated with general health, physical health, mental health, and diverse health behaviors.

**Methods:**

Study participants were drawn from a panel which is representative of the German-speaking population in Germany with Internet access. Participants answered a series of questionnaires regarding their climate change distress, climate change impairment, general health, physical health, mental health, and diverse health behaviors. To evaluate differences between subgroups, Bayesian independent samples t-tests were calculated. To evaluate associations between constructs, Bayesian correlations were calculated.

**Results:**

Especially women, younger people, people from West Germany, and people with a high level of formal education seem to experience higher levels of climate change distress. Regarding climate change impairment, the results suggest that especially women, older people, people from West Germany, people with a low level of formal education, people with a low or middle social status, and people with an inadequate/problematic health literacy seem to experience higher levels of climate change impairment. Furthermore, climate change distress and climate change impairment were weakly and differently associated with general health, physical health, mental health, and diverse health behaviors.

**Discussion:**

Climate change distress and impairment are not evenly distributed within German society. The results of this study provide a starting point for the development of target group specific intervention programs.

## 1 Introduction

Climate change has been widely recognized as one of the most challenging problems facing humanity and it imposes serious health threats (1–3). Furthermore, research suggest that climate change might have detrimental effects on mental health (4–6). Such findings are alarming because mental disorders are already a global problem that affect people from various age groups (7, 8). In Germany, for example, the 12-month prevalence of mental disorders is around 28% and only a minority of those affected seek adequate treatment (9–11). Globally, around one in eight people live with a mental disorder (12). Furthermore, data from the Global Burden of Disease study suggests that the mean prevalence of mental disorders is around 12% for individuals aged 5–24 years (13). Besides the individual suffering of those affected, mental disorders can cause enormous economic losses (14–16).

Researchers have used diverse measures to investigate how climate change and its perception relate to mental health (17–20). Recently, a promising measurement tool (18) was developed that differentiates between the affective experience of distress over climate change (climate change distress subscale, e.g., “When I think about climate change, I worry about the future”) and the functional impairments associated with climate change (climate change impairment subscale, e.g., “Because of climate change, I am overwhelmed by everyday activities”). Such a distinction is crucial because not all negative affective states are pathological, and they might even motivate pro-environmental behavior (18, 21, 22). Functional impairments, like not being able to work or maintaining social relationships, however, might require immediate treatment (18). While the items of the climate change distress subscale cover affective reactions like anxiety (e.g., “When I think about climate change, I worry about the future”), sadness (e.g., “I feel sad that climate change is causing people and animals to suffer”), and anger (e.g., “I feel angry when I see how little is done to combat climate change”), the items of the climate change impairment subscale address social relationships (e.g., “Constant discussions about climate change are affecting my relationships”), work performance (e.g., “When I think about climate change, I cannot bring myself to work/study”) and diverse other topics (18).

The present study assesses climate change distress and climate change impairment within the population of Germany using a population-representative sample. The results are meant to identify vulnerable subgroups within the population and thereby can help to facilitate the development of target group specific intervention programs that address climate change distress and climate change impairment. Additionally, the present study explores whether climate change distress and impairment are associated with health literacy, which refers to skills needed to adequately use health information when making health-relevant decisions (23). Since health literacy is associated with diverse health-related constructs and behaviors (24), it might be a relevant topic to cover within intervention programs that address climate change distress and impairment. Furthermore, to better understand the societal relevance of climate change distress and impairment and its association with health-related constructs, the present study explores whether climate change distress and climate change impairment are associated with general health, physical health, mental health, and diverse health behaviors (exercise routines, fruit consumption, vegetable consumption, soft drink consumption, alcohol consumption, and cigarette consumption).

## 2 Methods

### 2.1 Ethical considerations

The study was designed to comply with the ethical principles for medical research involving human subjects laid out in the Declaration of Helsinki. Before data collection, a study protocol was submitted to the ethics committee of the Berlin Medical Association. The ethics committee had no ethical or professional objections to the study protocol (reference number: Eth-64/23). Before the study started, all participants were informed about the content of the upcoming study, and they provided their informed consent to take part in the study. Furthermore, participants had the opportunity to opt out of the study at any time during the study.

### 2.2 Data acquisition

The German market research institute forsa Gesellschaft für Sozialforschung und statistische Analysen mbH (Forsa) was responsible for data acquisition. The survey was conducted using the online panel forsa.omninet, which is a representative panel of the German-speaking population in Germany with Internet access and has around 100,000 participants. A continuous recruiting process adds new participants to the panel every month. In addition, the composition of the panel is continuously monitored based on key characteristics (e.g., region, age, sex), and recruitment is adjusted accordingly. A random sample was drawn from all panel participants aged 16 years and older. Data acquisition took place from December 07 to December 19, 2023. This study was part of a larger study that assessed a variety of different constructs and health-related behaviors (e.g., social media use, eating behavior), and therefore the raw dataset contains additional variables that will not be described here because they exceed the scope of this study. Further analyses of the raw dataset with other thematic focuses are under way and will probably result in additional publications. For example, one analysis looks at eating behaviors and the corresponding manuscript is currently under review. The statistical analyses were based on the 2,969 study participants who answered all relevant items. Further information about the panel and data security measures can be found elsewhere (25, 26). Sample characteristics by sex, age, and state can be found in Table 1.

**Table 1:** Sample characteristics by sex, age, and state.

Variable	Subgroup	Frequency	Percent
Sex	Male	1,466	49.377
	Female	1,503	50.623
Age	16–29	527	17.750
	30–45	649	21.859
	46–64	935	31.492
	65+	858	28.899
State	Schleswig-Holstein	105	3.537
	Hamburg	77	2.593
	Niedersachsen	306	10.307
	Bremen	21	0.707
	Nordrhein-Westfalen	590	19.872
	Hessen	239	8.050
	Rheinland-Pfalz	166	5.591
	Baden-Württemberg	406	13.675
	Bayern	572	19.266
	Saarland	31	1.044
	Berlin	122	4.109
	Brandenburg	70	2.358
	Mecklenburg-Vorpommern	47	1.583
	Sachsen	113	3.806
	Sachsen-Anhalt	58	1.954
	Thüringen	46	1.549

### 2.3 Sample characteristics

A total of 2,969 study participants answered all relevant items. Sample characteristics by sex, age, and state can be found in Table 1.

### 2.4 Statistical analyses

Statistical analyses were conducted with JASP Version 0.18.3 (27). To evaluate differences between subgroups, Bayesian independent samples t-tests were calculated. Researchers interested in re-running the analyses can do so by applying the following specifications: Alternative Hypothesis (Group 1 ≠ Group 2), Bayes Factor (BF_10_), Tests (Student), Additional Statistics (Descriptives), Plots (Prior and posterior, Additional info, Credible interval 95.0%), Missing Values (Exclude cases per dependent variable), Prior (Default Cauchy scale 0.707).

To evaluate associations between constructs, Bayesian correlations were calculated. Researchers interested in re-running the analyses can do so by applying the following specifications: Population Correlation Coefficient (Pearson’s rho), Alternative Hypothesis (Correlated), Bayes Factor (BF_10_), Additional Options (Display pairwise table, Report Bayes factors, Flag supported correlations, Sample size, Credible intervals 95.0%), Prior (Stretched beta prior width 1.0).

### 2.5 Measures

Climate change distress and climate change impairment were assessed with the German version of the climate change distress and impairment scale (18). The instrument consists of 23 items that comprise the two subscales climate change distress (15 items: e.g., “When I think about climate change, I worry about the future”) and climate change impairment (8 items: e.g., “Because of climate change, I am overwhelmed by everyday activities”). Participants rated all items on scales ranging from strongly disagree (1), disagree (2), neutral (neither agree nor disagree) (3), agree (4), to strongly agree (5). After recoding the negative phrased items, scores were generated for each subscale by calculating the means. Bayesian scale reliability statistics were calculated for the climate change distress (Cronbach’s α posterior mean = 0.916 [95% credible interval: 0.912, 0.921]) and climate change impairment subscales (Cronbach’s α posterior mean = 0.769 [95% credible interval: 0.755, 0.781]).

General, physical and mental health were assessed with 2 questions that were drawn from previous nationally representative studies and a third adapted question (28, 29). The questions addressed general health (“Overall, how do you currently rate your general health?”), physical health (“Overall, how do you currently rate your physical health?”), and mental health (“Overall, how do you currently rate your mental health?”). The questions were answered on scales ranging from 0 (very bad) to 10 (very good).

Health behaviors were assessed with 6 questions that were drawn from previous nationally representative studies (28, 29). Participants answered questions about their health behaviors within a typical week. The questions addressed exercise routines (“On average, how many days a week do you exercise?”), fruit consumption (“On average, how many days a week do you eat fruit?”), vegetable consumption (“On average, how many days a week do you eat vegetables?”), soft drink consumption (“On average, how many days a week do you drink sugary soft drinks?”), alcohol consumption (“On average, how many days a week do you drink alcohol?”), and cigarette consumption (“On average, how many days a week do you smoke cigarettes?”). The questions were answered on scales ranging from 0 (0 days) to 7 (7 days).

### 2.6 Subgroups

To identify vulnerable subgroups, participants were classified along the lines of sex (male, female), age (16–29 years, 30–64 years, 65+ years), region (West Germany + Berlin, East Germany), education (low, middle, high), social status (low, middle, high), migration background (no, yes), and health literacy (inadequate/problematic, sufficient). Demographic information of study participants (e.g., sex, age, region) was provided by the market research institute forsa Gesellschaft für Sozialforschung und statistische Analysen mbH (Forsa). Until German reunification in 1990, Germany was divided into the Federal Republic of Germany (predominantly located in the western part of today’s Germany) and the German Democratic Republic (predominantly located in the eastern part of today’s Germany). As some cultural differences between the regions may still exist today, region was included as a subgroup classification factor in the analyses.

Education was assessed by asking people about their highest educational qualification. Participants were classified as possessing a low (roughly equivalent to no formal education or basic secondary school: ohne Haupt-/Volksschulabschluss; Haupt-/Volksschulabschluss), middle (roughly equivalent to intermediate secondary school: Mittlere Reife, Realschulabschluss, Fachschulreife; Abschluss der Polytechnischen Oberschule; Fachhochschulreife, Abschluss einer Fachoberschule), or high formal level of education (roughly equivalent to most advanced secondary school, e.g., grammar schools to obtain a general or specialized university entrance qualification, or university degree: Abitur, allgemeine oder fachgebundene Hochschulreife; Fach-/Hochschulstudium).

Social status was assessed with the German version of the MacArthure Scale (30). Participants rated their subjective social status on a scale ranging from 1 (lowest rating) to 10 (highest rating). Participants were classified as possessing a low (scores: 1–4), middle (scores: 5–7), or high subjective social status (scores: 8–10).

Health literacy was assessed with the European Health Literacy Survey Q16 instrument (31, 32). The instrument consists of 16 items (e.g., “On a scale from very easy to very difficult, how easy would you say it is to find information on treatments of illnesses that concern you?”). Participants rated all items on scales ranging from (1) very easy, (2) easy, (3) difficult, to (4) very difficult. Answers were dichotomized (1 = very easy/easy, 0 = difficult/very difficult) and added up to create a score that allows to differentiate between people with inadequate/problematic health literacy (scores: 0–12) and people with sufficient health literacy (scores: 13–16). Bayesian scale reliability statistics were calculated (Cronbach’s α posterior mean = 0.818 [95% credible interval: 0.809, 0.827]).

## 3 Results

### 3.1 Climate change distress

In order to evaluate the relevance of climate change distress and impairment within society, it can be helpful to look at how many people (strongly) agree or (strongly) disagree with specific items. More than 48% of the study participants report that they feel angry when they see how little is done to combat climate change. More than 62% report that when they think about climate change, they worry about the future. More than 57% report that they are enraged that we have missed many chances to stop climate change. More than 49% report that news about climate change make them feel depressed. More than 43% report that the uncertainty about how climate change will progress scares them. More than 69% report that they feel sad that climate change is causing people and animals to suffer. More than 49% report that they are scared that people will lose their homes because of climate change. More than 67% report that they feel sad that some parts of the environment will not recover from the effects of climate change. And more than 59% report that the impact that climate change has had on the planet saddens them.

Less than 20% of the study participants report that they are not sad about climate change. Less than 34% report that they do not fear for their future on this planet. Less than 19% report that they are not mad when others damage the climate. Less than 30% report that they do not get upset when others ignore climate change. Less than 23% report that they are not angry that some countries have missed their climate protection goals. And less than 10% report that they feel carefree when they think about climate change. Detailed information about the answers to the climate change distress subscale can be found in Table 2.

**Table 2:** Climate change distress of the population in Germany: Answers to the climate change distress subscale.

Nr.	Item	Strongly disagree (stimme überhaupt nicht zu)	Disagree (stimmte nicht zu)	Neutral (neither agree nor disagree) (neutral [weder Zustimmung noch Ablehnung])	Agree (stimme zu)	Strongly agree (stimme voll und ganz zu)
1	I feel angry when I see how little is done to combat climate change. (Ich bin wütend, wenn ich sehe, wie wenig gegen den Klimawandel getan wird.)	11.0% (326)	12.3% (365)	28.6% (848)	32.6% (968)	15.6% (462)
2	When I think about climate change, I worry about the future. (Wenn ich an den Klimawandel denke, sorge ich mich um die Zukunft.)	8.2% (244)	11.2% (333)	18.0% (533)	45.3% (1345)	17.3% (514)
3	I am not sad about climate change. (Ich bin nicht traurig über den Klimawandel.) (r)	24.9% (740)	30.5% (906)	24.7% (732)	12.9% (382)	7.0% (209)
4	I am enraged that we have missed many chances to stop climate change. (Ich bin wütend darüber, dass wir viele Chancen verpasst haben, den Klimawandel zu stoppen.)	9.0% (266)	10.0% (296)	23.6% (700)	36.1% (1072)	21.4% (635)
5	I do not fear for my future on this planet. (Ich habe keine Angst um meine Zukunft auf diesem Planeten.) (r)	12.4% (369)	27.7% (822)	26.6% (790)	23.6% (700)	9.7% (288)
6	News about climate change makes me feel depressed. (Nachrichten über den Klimawandel bedrücken mich.)	9.6% (284)	13.3% (394)	27.3% (812)	40.2% (1194)	9.6% (285)
7	I am not mad when others damage the climate. (Ich bin nicht sauer, wenn andere dem Klima schaden.) (r)	21.5% (637)	37.3% (1106)	22.6% (670)	13.4% (399)	5.3% (157)
8	The uncertainty about how climate change will progress scares me. (Die Ungewissheit darüber, wie der Klimawandel voranschreiten wird, macht mir Angst.)	12.0% (355)	16.7% (497)	27.9% (828)	34.4% (1022)	9.0% (267)
9	I feel sad that climate change is causing people and animals to suffer. (Ich bin traurig darüber, dass Menschen und Tiere unter dem Klimawandel leiden.)	4.9% (144)	5.5% (163)	20.2% (601)	48.3% (1434)	21.1% (627)
10	I do not get upset when others ignore climate change. (Ich rege mich nicht auf, wenn andere den Klimawandel ignorieren.) (r)	14.1% (420)	30.9% (917)	25.9% (770)	18.6% (552)	10.4% (310)
11	I am scared that people will lose their homes because of climate change. (Ich habe Angst davor, dass Menschen durch den Klimawandel ihr zu Hause verlieren.)	8.5% (251)	13.1% (389)	29.2% (866)	39.1% (1161)	10.2% (302)
12	I feel sad that some parts of the environment will not recover from the effects of climate change. (Ich bin traurig darüber, dass sich manche Teile der Umwelt nicht mehr von den Auswirkungen des Klimawandels erholen werden.)	6.1% (180)	6.2% (185)	20.7% (614)	45.7% (1358)	21.3% (632)
13	I am not angry that some countries have missed their climate protection goals. (Ich bin nicht wütend darüber, dass manche Länder ihre Klimaschutzziele verfehlt haben.) (r)	18.6% (553)	32.0% (950)	26.5% (788)	15.1% (449)	7.7% (229)
14	The impact that climate change has had on the planet saddens me. (Die Auswirkungen, die der Klimawandel auf den Planeten hat, machen mich traurig.)	7.8% (232)	9.4% (279)	23.7% (704)	41.7% (1238)	17.4% (516)
15	I feel carefree when I think about climate change. (Ich fühle mich unbeschwert, wenn ich an den Klimawandel denke.) (r)	26.5% (787)	37.6% (1115)	26.3% (780)	5.6% (167)	4.0% (120)

The items come from an article (18) that is licensed under a Creative Commons Attribution 4.0 International License (https://creativecommons.org/licenses/by/4.0/).

### 3.2 Climate change impairment

More than 2% of the study participants report that climate change drains all their energy. More than 9% report that when they think about climate change, they get a headache or stomachache. More than 2% report that because of climate change, they are overwhelmed by everyday activities. More than 6% report that constant discussions about climate change are affecting their relationships. More than 2% report that when they think about climate change, they cannot bring themselves to work/study.

Less than 72% of the study participants report that their thoughts and feelings about climate change do not affect how well they sleep. Less than 54% report that their thoughts and feelings about climate change do not negatively impact their everyday life. And less than 38% report that they have no trouble mentally tuning out climate change. Detailed information about the answers to the climate change impairment subscale can be found in Table 3.

**Table 3:** Climate change impairment of the population in Germany: Answers to the climate change impairment subscale.

Nr.	Item	Strongly disagree (stimme überhaupt nicht zu)	Disagree (stimmte nicht zu)	Neutral (neither agree nor disagree) (neutral [weder Zustimmung noch Ablehnung])	Agree (stimme zu)	Strongly agree (stimme voll und ganz zu)
16	Climate change drains all my energy. (Der Klimawandel raubt mir alle Energie.)	46.7% (1388)	33.5% (995)	16.8% (499)	2.5% (75)	0.4% (12)
17	My thoughts and feelings about climate change do not affect how well I sleep. (Meine Gedanken und Gefühle zum Klimawandel beeinflussen nicht, wie gut ich schlafe.) (r)	5.8% (173)	7.3% (216)	15.8% (469)	34.6% (1026)	36.5% (1085)
18	When I think about climate change, I get a headache or stomachache. (Wenn ich über den Klimawandel nachdenke, bekomme ich Kopf- oder Bauchschmerzen.)	40.5% (1203)	29.7% (882)	20.3% (603)	7.8% (231)	1.7% (50)
19	Because of climate change, I am overwhelmed by everyday activities. (Wegen des Klimawandels bin ich vom Alltag überfordert.)	45.6% (1353)	35.9% (1066)	15.7% (467)	2.2% (66)	0.6% (17)
20	My thoughts and feelings about climate change do not negatively impact my everyday life. (Meine Gedanken und Gefühle zum Klimawandel beeinträchtigen meinen Alltag nicht.) (r)	6.0% (177)	15.6% (463)	24.8% (735)	35.1% (1041)	18.6% (553)
21	I have no trouble mentally tuning out climate change. (Ich habe keine Schwierigkeiten damit, den Klimawandel gedanklich auszublenden.) (r)	7.4% (220)	25.0% (741)	30.4% (903)	25.1% (745)	12.1% (360)
22	Constant discussions about climate change are affecting my relationships. (Ständige Diskussionen über den Klimawandel beeinträchtigen meine Beziehungen.)	39.1% (1162)	34.4% (1020)	19.6% (583)	5.5% (164)	1.3% (40)
23	When I think about climate change, I cannot bring myself to work/study. (Wenn ich an den Klimawandel denke, kann ich mich nicht aufraffen, zu arbeiten/lernen.)	54.3% (1611)	29.6% (878)	14.0% (415)	1.6% (48)	0.6% (17)

The items come from an article (18) that is licensed under a Creative Commons Attribution 4.0 International License (https://creativecommons.org/licenses/by/4.0/).

### 3.3 Sex

Males showed lower climate change distress (extreme evidence for H_1_: BF_10_ = 1.196 × 10^+28^; error % = 2.373 × 10^−30^; effect size δ = −0.432 [95% credible interval: −0.504, −0.359]) and lower climate change impairment (extreme evidence for H_1_: BF_10_ = 5.634 × 10^+8^; error % = 4.364 × 10^−11^; effect size δ = −0.251 [95% credible interval: −0.323, −0.179]) than females.

### 3.4 Age

People under the age of 30 years showed higher climate change distress than people aged 30–64 years (extreme evidence for H_1_: BF_10_ = 4.103 × 10^+12^; error % = 6.502 × 10^−15^; effect size δ = 0.403 [95% credible interval: 0.304, 0.503]) and people aged 65 years and older (extreme evidence for H_1_: BF_10_ = 1772.462; error % = 1.341 × 10^−5^; effect size δ = 0.250 [95% credible interval: 0.142, 0.359]). People 30–64 years showed lower climate change distress than people aged 65 years and older (extreme evidence for H_1_: BF_10_ = 717.869; error % = 3.240 × 10^−5^; effect size δ = −0.186 [95% credible interval: −0.269, −0.103]).

People under the age of 30 years showed higher climate change impairment than people aged 30–64 years (anecdotal evidence for H_1_: BF_10_ = 1.726; error % = 0.013; effect size δ = 0.131 [95% credible interval: 0.033, 0.229]) and lower climate change impairment than people aged 65 years and older (anecdotal evidence for H_1_: BF_10_ = 1.287; error % = 0.017; effect size δ = −0.136 [95% credible interval: −0.244, −0.028]). People aged 30–64 years showed lower climate change impairment than people aged 65 years and older (extreme evidence for H_1_: BF_10_ = 5.432 × 10^+7^; error % = 4.541 × 10^−10^; effect size δ = −0.274 [95% credible interval: −0.357, −0.191]).

### 3.5 Region

People from West Germany showed higher climate change distress (extreme evidence for H_1_: BF_10_ = 34314.764; error % = 6.834 × 10^−7^; effect size δ = 0.297 [95% credible interval: 0.184, 0.411]) and climate change impairment (strong evidence for H_1_: BF_10_ = 29.136; error % = 7.572 × 10^−4^; effect size δ = 0.202 [95% credible interval: 0.089, 0.315]) than people from East Germany.

### 3.6 Education

People with a lower level of formal education and people with a middle level of formal education did not seem to differ regarding climate change distress (moderate evidence for H_0_: BF_10_ = 0.171; error % = 0.128; effect size δ = 0.072 [95% credible interval: −0.020, 0.164]). People with a lower level of formal education showed lower climate change distress than people with a high level of formal education (extreme evidence for H_1_: BF_10_ = 11620.311; error % = 2.052 × 10^−6^; effect size δ = −0.234 [95% credible interval: −0.326, −0.141]). People with a middle level of formal education showed lower climate change distress than people with a high level of formal education (extreme evidence for H_1_: BF_10_ = 1.715 × 10^+9^; error % = 1.474 × 10^−11^; effect size δ = −0.297 [95% credible interval: −0.381, −0.213]).

People with a lower level of formal education showed higher climate change impairment than people with a middle level of formal education (extreme evidence for H_1_: BF_10_ = 221.742; error % = 1.044 × 10^−4^; effect size δ = 0.193 [95% credible interval: 0.100, 0.285]) and people with a high level of formal education (extreme evidence for H_1_: BF_10_ = 470.386; error % = 4.949 × 10^−5^; effect size δ = 0.201 [95% credible interval: 0.108, 0.293]). People with a middle level of formal education and people with a high level of formal education did not seem to differ regarding climate change impairment (strong evidence for H_0_: BF_10_ = 0.049; error % = 0.445; effect size δ = 0.011 [95% credible interval: −0.072, 0.094]).

### 3.7 Social status

People with a low social status did not seem to differ from people with a middle social status (strong evidence for H_0_: BF_10_ = 0.070; error % = 0.300; effect size δ = 0.029 [95% credible interval: −0.077, 0.134]) and people with a high social status (anecdotal evidence for H_0_: BF_10_ = 0.730; error % = 0.029; effect size δ = 0.137 [95% credible interval: 0.012, 0.262]) regarding climate change distress. People with a middle social status showed higher climate change distress than people with a high social status (anecdotal evidence for H_1_: BF_10_ = 1.421; error % = 0.016; effect size δ = 0.120 [95% credible interval: 0.028, 0.212]).

People with a low social status and people with a middle social status did not seem to differ regarding climate change impairment (moderate evidence for H_0_: BF_10_ = 0.163; error % = 0.130; effect size δ = 0.076 [95% credible interval: −0.030, 0.182]). People with a low social status showed higher climate change impairment than people with a high social status (extreme evidence for H_1_: BF_10_ = 45228.701; error % = 5.649 × 10^−7^; effect size δ = 0.332 [95% credible interval: 0.206, 0.458]). People with a middle social status showed higher climate change impairment than people with a high social status (extreme evidence for H_1_: BF_10_ = 444288.204; error % = 5.395 × 10^−8^; effect size δ = 0.265 [95% credible interval: 0.173, 0.357]).

### 3.8 Migration background

People without a migration background and people with a migration background did not seem to differ regarding climate change distress (strong evidence for H_0_: BF_10_ = 0.085; error % = 0.219; effect size δ = −0.008 [95% credible interval: −0.156, 0.139]) and climate change impairment (strong evidence for H_0_: BF_10_ = 0.093; error % = 0.201; effect size δ = 0.032 [95% credible interval: −0.115, 0.180]).

### 3.9 Health literacy

People with inadequate/problematic health literacy and people with sufficient health literacy did not seem to differ regarding climate change distress (anecdotal evidence for H_0_: BF_10_ = 0.699; error % = 0.032; effect size δ = 0.089 [95% credible interval: 0.015, 0.162]). However, people with inadequate/problematic health literacy showed higher climate change impairment than people with sufficient health literacy (extreme evidence for H_1_: BF_10_ = 3.111 × 10^+7^; error % = 7.813 × 10^−10^; effect size δ = 0.240 [95% credible interval: 0.166, 0.313]). Further information on climate change distress of the population in Germany by sex, age, region, education, social status, migration background, and health literacy can be found in Table 4. Further information on climate change impairment of the population in Germany by sex, age, region, education, social status, migration background, and health literacy can be found in Table 5.

**Table 4:** Climate change distress of the population in Germany by sex, age, region, education, social status, migration background, and health literacy.

Category	Group	N	Mean	SD	SE	Coefficient of variation	Lower 95% CI	Upper 95% CI
Population in Germany	Overall	2,969	3.435	0.769	0.014	0.224	3.407	3.462
Sex	Male	1,466	3.270	0.806	0.021	0.246	3.229	3.311
	Female	1,503	3.596	0.694	0.018	0.193	3.560	3.631
Age	<30 years	527	3.658	0.753	0.033	0.206	3.593	3.722
	30–64 years	1,584	3.337	0.802	0.020	0.240	3.297	3.376
	65+ years	858	3.479	0.678	0.023	0.195	3.433	3.524
Region	West Germany (incl. Berlin)	2,635	3.461	0.753	0.015	0.218	3.432	3.489
	East Germany	334	3.231	0.858	0.047	0.265	3.138	3.323
Education	Low	754	3.388	0.713	0.026	0.211	3.337	3.439
	Middle	1,100	3.334	0.772	0.023	0.231	3.288	3.379
	High	1,115	3.566	0.784	0.023	0.220	3.520	3.612
Social Status	Low	411	3.472	0.822	0.041	0.237	3.392	3.552
	Middle	1966	3.450	0.743	0.017	0.215	3.417	3.483
	High	592	3.358	0.810	0.033	0.241	3.293	3.424
Migration background	No	2,785	3.434	0.770	0.015	0.224	3.406	3.463
	Yes	184	3.441	0.751	0.055	0.218	3.332	3.550
Health literacy	Inadequate/Problematic	1,187	3.476	0.749	0.022	0.215	3.433	3.518
	Sufficient	1782	3.407	0.781	0.018	0.229	3.371	3.444

**Table 5:** Climate change impairment of the population in Germany by sex, age, region, education, social status, migration background, and health literacy.

Category	Group	N	Mean	SD	SE	Coefficient of variation	Lower 95% CI	Upper 95% CI
Population in Germany	Overall	2,969	2.088	0.617	0.011	0.296	2.066	2.110
Sex	Male	1,466	2.010	0.612	0.016	0.304	1.978	2.041
	Female	1,503	2.164	0.613	0.016	0.283	2.133	2.195
Age	<30 years	527	2.108	0.659	0.029	0.313	2.052	2.165
	30–64 years	1,584	2.025	0.622	0.016	0.307	1.994	2.056
	65+ years	858	2.191	0.566	0.019	0.258	2.153	2.229
Region	West Germany (incl. Berlin)	2,635	2.102	0.620	0.012	0.295	2.078	2.126
	East Germany	334	1.976	0.585	0.032	0.296	1.913	2.039
Education	Low	754	2.179	0.612	0.022	0.281	2.135	2.222
	Middle	1,100	2.060	0.609	0.018	0.296	2.024	2.096
	High	1,115	2.054	0.622	0.019	0.303	2.017	2.090
Social status	Low	411	2.161	0.670	0.033	0.310	2.096	2.226
	Middle	1966	2.113	0.610	0.014	0.289	2.086	2.140
	High	592	1.952	0.584	0.024	0.299	1.905	1.999
Migration background	No	2,785	2.089	0.611	0.012	0.293	2.066	2.112
	Yes	184	2.069	0.701	0.052	0.339	1.967	2.171
Health literacy	Inadequate/Problematic	1,187	2.176	0.630	0.018	0.290	2.140	2.212
	Sufficient	1782	2.029	0.601	0.014	0.296	2.001	2.057

### 3.10 Correlation analyses

Climate change distress was weakly negatively correlated with mental health, soft drink consumption, alcohol consumption, and cigarette consumption. Furthermore, climate change distress was weakly positively correlated with fruit consumption and vegetable consumption. Further information on the associations between climate change distress, health status, and health behaviors can be found in Table 6.

**Table 6:** Associations between climate change distress, health status, and health behaviors.

Category	Variable 1	Variable 2	n	Pearson’s r	BF_10_	Lower 95% CI	Upper 95% CI
Health status							
	Climate change distress	General health	2,969	−0.030	0.086	−0.066	0.006
	Climate change distress	Physical health	2,969	−0.043	0.343	−0.078	−0.007
	Climate change distress	Mental health	2,969	−0.121***	8.497 × 10^+7^	−0.157	−0.086
Health behaviors							
	Climate change distress	Exercise routines	2,969	0.049	0.850	0.013	0.085
	Climate change distress	Fruit consumption	2,969	0.100***	71986.475	0.064	0.136
	Climate change distress	Vegetable consumption	2,969	0.165***	1.504 × 10^+16^	0.130	0.200
	Climate change distress	Soft drink consumption	2,969	−0.104***	255708.615	−0.140	−0.069
	Climate change distress	Alcohol consumption	2,969	−0.068*	22.859	−0.104	−0.032
	Climate change distress	Cigarette consumption	2,969	−0.068*	20.913	−0.103	−0.032

*BF_10_ > 10, **BF_10_ > 30, ***BF_10_ > 100.

Climate change impairment was weakly negatively correlated with general health, physical health, and mental health. Furthermore, climate change impairment was weakly positively correlated with fruit consumption and vegetable consumption. Further information on the associations between climate change impairment, health status, and health behaviors can be found in Table 7.

**Table 7:** Associations between climate change impairment, health status and health behaviors.

Category	Variable 1	Variable 2	n	Pearson’s r	BF_10_	Lower 95% CI	Upper 95% CI
Health status							
	Climate change impairment	General health	2,969	−0.186***	8.819 × 10^+20^	−0.220	−0.151
	Climate change impairment	Physical health	2,969	−0.162***	3.205 × 10^+15^	−0.197	−0.127
	Climate change impairment	Mental health	2,969	−0.262***	1.963 × 10^+44^	−0.295	−0.228
Health behaviors							
	Climate change impairment	Exercise routines	2,969	0.046	0.523	0.010	0.082
	Climate change impairment	Fruit consumption	2,969	0.108***	747893.615	0.072	0.143
	Climate change impairment	Vegetable consumption	2,969	0.066*	15.596	0.030	0.102
	Climate change impairment	Soft drink consumption	2,969	−0.030	0.090	−0.066	0.006
	Climate change impairment	Alcohol consumption	2,969	−0.050	0.992	−0.086	−0.014
	Climate change impairment	Cigarette consumption	2,969	−0.040	0.241	−0.076	−0.004

*BF_10_ > 10, **BF_10_ > 30, ***BF_10_ > 100.

## 4 Discussion

### 4.1 Principal findings

This study assessed climate change distress (e.g., affective experience of distress over climate change) and climate change impairment (e.g., functional impairments associated with climate change) within the population of Germany using a population-representative sample (18). The results identify vulnerable subgroups and thereby can help to facilitate the development of target group specific intervention programs. Furthermore, this study explored whether climate change distress and climate change impairment are associated with general health, physical health, mental health, and diverse health behaviors.

Overall, the results demonstrate that climate change distress and climate change impairment are not evenly distributed within German society. Especially women, younger people, people from West Germany, and people with a high level of formal education seem to experience higher levels of climate change distress. Do these results align with previous findings? In some respects, the answer seems to be yes. Previous research, for example, suggests that younger people and women are more likely to be distressed about climate change (19). Furthermore, research suggest that women are generally more likely than men to report psychological distress, which might be explained by the interaction of diverse factors and social circumstances (33–35). But why should people from West Germany and people with a high level of formal education experience higher levels of climate change distress? Previous research suggests that people from East Germany are more skeptical about climate change and that people with a college degree are less skeptical about climate change (36). Therefore, one might speculate that people who are more skeptical about climate change would also be less inclined to experience climate change distress. Compared to the results of another smaller German speaking sample (M = 3.73) reported elsewhere (18), the participants of the current study (M = 3.435) showed slightly lower climate change distress levels.

Turning to climate change impairment, the results suggest that especially women, older people, people from West Germany, people with a low level of formal education, people with a low or middle social status, and people with an inadequate/problematic health literacy seem to experience higher levels of climate change impairment. Do these results also align with previous findings from other fields of research? Again, regarding many findings, the answer seems to be yes. As a reminder, functional impairments, like not being able to work or maintaining social relationships, might require immediate treatment (18). Therefore, instead of comparing the climate change impairment results to results from psychological distress research, it seems more adequate to compare them to findings from more general areas of mental health research. In line with the results of this study, previous research suggest that women show a higher 12-month prevalence of mental disorders than men (9–11). Furthermore, women seem to be more likely to report physical and somatoform symptoms and they show a higher point prevalence of depression (37, 38). Concerning education, a previous study found that an additional year of education was associated with a lower likelihood of reporting symptoms related to anxiety and depression (39). Furthermore, a previous study suggests that there is an inversed association between subjective social status and diverse mental disorders (40). Regarding health literacy, previous studies suggest that mental health literacy and digital health literacy are positively associated with mental health (28, 29). But why should older people and people from West Germany experience higher levels of climate change impairment? Overall rates of mental disorders are typically higher in younger people than older people (9–11). However, one might speculate that older people experience more climate change impairment because they know that older people are typically more affected by heat-related illnesses like classical heatstroke (41). Regarding the regional divide, one might again speculate that people from East Germany are more skeptical about climate change and therefore less likely to ruminate about climate change and less inclined to experience climate change impairment (36). Compared to the results of another smaller German speaking sample (M = 1.96) reported elsewhere (18), the participants of the current study (M = 2.088) showed slightly higher climate change impairment levels.

Regarding age, it is worth noting that younger and older people seem to be more affected by climate change distress and impairment than middle-aged people. As one reviewer of this article pointed out, it would be interesting to explore whether different mechanism could be responsible for this result. For example, climate anxiety seems to be more pronounced in younger people and therefore might be one explanation for the elevated levels of climate change distress and impairment in younger people (22, 42). Furthermore, one might speculate that solastalgia, which refers to distress caused by changes in the environment, could be responsible for elevated levels of climate change distress and impairment in older people (43).

What should be the next steps considering the results of this study? The answer to this question depends on the chosen long-term goal. If relieving distress is the goal, one might want to build intervention programs that focus on women, young people, people from West Germany, and people with a high level of formal education because they seem to experience higher levels of climate change distress. If the goal is to motivate people to engage in pro-environmental behavior, however, one might take a different path. In the introduction, it has been mentioned that not all negative affective states are pathological, and that they might motivate pro-environmental behavior (18, 21, 22). Therefore, one might want to start their endeavor of motivating pro-environmental behavior by focusing on people who do not experience high levels of climate change distress. Regarding climate change impairment, the question will probably be easier to answer because many people will agree that functional impairment might require immediate treatment and that relieving suffering is a noble goal (18). Therefore, one might want to start building climate change impairment intervention programs that focus on women, older people, people from West Germany, people with a low level of formal education, people with a low or middle social status, and people with an inadequate/problematic health literacy.

Besides identifying vulnerable subgroups who would benefit from target group specific intervention programs, associations between climate change distress, climate change impairment, diverse health behaviors, and health-related constructs were explored. Climate change distress was negatively correlated with mental health, soft drink consumption, alcohol consumption, and cigarette consumption. Furthermore, climate change distress was positively correlated with fruit consumption and vegetable consumption. That people with higher climate change distress rate their mental health more negatively seems hardly surprising. Furthermore, one might speculate that people who experience higher climate change distress are more concerned about the environment and health in general and therefore express better health behaviors in their daily lives. It needs to be stressed, however, that the found associations were very weak and therefore should not be overinterpreted.

Climate change impairment was negatively correlated with general health, physical health, and mental health. Furthermore, climate change impairment was positively correlated with fruit consumption and vegetable consumption. If climate change impairment is interpreted as a state that might require treatment, it seems logical that it is negatively correlated with the general, physical, and mental health of a person. Furthermore, one might speculate that people with higher climate change impairment try to better their situation by expressing more healthy eating behaviors. But again, it needs to be stressed that the found associations were very weak and therefore should not be overinterpreted. Furthermore, when interpreting the results, it is important to keep in mind that simple correlations, instead of partial or multiple correlations, were calculated.

Before turning to the limitations and future directions, it seems sensible to take a step back and look at some responses that the study participants gave, and which can be found in Tables 2, 3. Regarding climate change distress, more than 62% of the study participants report that when they think about climate change, they worry about the future, and more than 49% report that news about climate change make them feel depressed. Regarding climate change impairment, more than 9% of the study participants report that when they think about climate change, they get a headache or stomachache, and more than 6% report that constant discussions about climate change are affecting their relationships. These figures are remarkable because they demonstrate that climate change distress and climate change impairment are highly relevant topics within society with real world implications for many people.

### 4.2 Limitations and future directions

There are various limitations that must be kept in mind when interpreting the results of this study. Three limitations seem especially important and will therefore be discussed in greater detail. The first limitation concerns the chosen study design. Because a cross-sectional study design was chosen, no causal inferences can be drawn (44). What does that mean? This study found, for example, that climate change distress is positively correlated with vegetable consumption. Some people might be inclined to conclude that climate change distress causes people to eat more vegetables (or vice versa). This, however, would be an unjustified conclusion. To draw such a causal inference, future studies would need to design a controlled experiment in which they manipulate peoples’ climate change distress and afterwards measure their fruit consumption (45).

The second limitation concerns the chosen data collection method. As described in the methods section, the survey was conducted using the online panel forsa.omninet, which is a representative panel of the German-speaking population in Germany with Internet access, and participants gave their answers using a web-based survey platform. This implies that basic computer skills and an Internet connection were necessary to take part in the study. Previous studies have found, however, that younger people use the Internet more frequently than older people (46). Therefore, when interpreting the results of this study, one must bear in mind that the results can only be representative of the German-speaking population in Germany with Internet access and basic computer skills. To overcome this limitation, future studies could use other data collection methods like face-to-face interviews. Even though the study sample was taken from a representative panel, the sample characteristics in Table 1 show that the study sample cannot be fully representative of the German-speaking population in Germany. In 2023, for example, the state of Sachsen had slightly more inhabitants than the state of Berlin (47). In the study sample, however, slightly more study participants came from the state of Berlin than from the state of Sachsen. Even though this difference might seem small, future studies could repeat the current analyses with survey weights to correct for such differences. The third limitation concerns the chosen types of measures. Besides using multi-item measures like the German version of the climate change distress and impairment scale (CC-DIS) (18), this study also relied on single-item measures. Even though previous research has found that single-item measures can be reliable and valid, one might argue that single-item measures are not ideal for measuring complex constructs (48–52). To overcome this limitation, future studies could replace the single-item measures with multi-item measures and examine whether the results would change in a significant way.

Furthermore, as one reviewer of this article pointed out, the current study mainly focuses on simple group comparisons and correlations. In future analyses, it would be interesting to conduct multivariate analyses that can control for key variables and explore whether certain factors (e.g., potentially modifiable factors such as health literacy) might influence or mediate the found relationships. Since those who report climate change distress are not necessarily the same as those who report climate change impairment, future studies could focus on exploring whether certain factors (e.g., education, social status) can function as impairment buffers.

## 5 Conclusion

This study suggests that climate change distress and climate change impairment are unevenly distributed within society. Furthermore, the results of this study can be used as a starting point for the development of target group specific intervention programs.

## Data Availability

The datasets presented in this article are not readily available because the datasets generated and analyzed during this study are the property of the independent, nonprofit foundation Stiftung Gesundheitswissen and are available on reasonable request. Requests to access the datasets should be directed to lars.koenig@stiftung-gesundheitswissen.de.
